# No effect of creatine supplementation on oxidative stress and cardiovascular parameters in spontaneously hypertensive rats

**DOI:** 10.1186/1550-2783-9-13

**Published:** 2012-04-05

**Authors:** Christiano RR Alves, Igor H Murai, Pamella Ramona, Humberto Nicastro, Luiz RG Bechara, Antonio H Lancha, Patrícia C Brum, Maria C Irigoyen, Bruno Gualano

**Affiliations:** 1 School of Physical Education and Sports, University of São Paulo, Av. Prof. Mello Moraes, 65, São Paulo, PO Box 05508-030, Brazil; 2 Hypertension Unit of Heart Institute, Medical School, University of São Paulo, São Paulo, Brazil

**Keywords:** Arterial hypertension, Therapeutic effects, Phosphocreatine

## Abstract

**Background:**

Exacerbated oxidative stress is thought to be a mediator of arterial hypertension. It has been postulated that creatine (Cr) could act as an antioxidant agent preventing increased oxidative stress. The aim of this study was to investigate the effects of nine weeks of Cr or placebo supplementation on oxidative stress and cardiovascular parameters in spontaneously hypertensive rats (SHR).

**Findings:**

Lipid hydroperoxidation, one important oxidative stress marker, remained unchanged in the coronary artery (Cr: 12.6 ± 1.5 vs. Pl: 12.2 ± 1.7 nmol·mg^-1^; p = 0.87), heart (Cr: 11.5 ± 1.8 vs. Pl: 14.6 ± 1.1 nmol·mg^-1^; p = 0.15), plasma (Cr: 67.7 ± 9.1 vs. Pl: 56.0 ± 3.2 nmol·mg^-1^; p = 0.19), plantaris (Cr: 10.0 ± 0.8 vs. Pl: 9.0 ± 0.8 nmol·mg^-1^; p = 0.40), and EDL muscle (Cr: 14.9 ± 1.4 vs. Pl: 17.2 ± 1.5 nmol·mg^-1^; p = 0.30). Additionally, Cr supplementation affected neither arterial blood pressure nor heart structure in SHR (p > 0.05).

**Conclusions:**

Using a well-known experimental model of systemic arterial hypertension, this study did not confirm the possible therapeutic effects of Cr supplementation on oxidative stress and cardiovascular dysfunction associated with arterial hypertension.

## Introduction

It has been suggested that exacerbated oxidative stress and its consequent oxidative damage may be mediators involved in cardiovascular diseases, such as systemic arterial hypertension [1]. Supporting this notion, a reduction in antioxidant bioavailability along with increased oxidative stress has been reported in both experimental and human hypertension [2].

Creatine (Cr) supplementation has emerged as a promising adjunct therapy in several pathological conditions [3], including cardiovascular diseases [4,5]. Interestingly, a growing body of experimental and clinical literature has suggested that Cr may exert protective effect in diseases where exacerbated oxidative stress plays a detrimental role (e.g., Huntington's disease) [6-8]. In fact, in vitro experiments have revealed that Cr may possess antioxidant properties by acting as a scavenger of free radicals, such as superoxide anions and peroxynitrite [8,9]. For instance, Cr pre-loading was found to be cytoprotective in different cell cultures with oxidative stressors (i.e., H2O2, tBOOH and peroxynitrite) [10]. Moreover, Cr may also "indirectly" attenuate the formation of reactive oxygen species trough the coupling of Cr with ATP into the mitochondria, ultimately resulting in a more efficient mitochondrial respiration and delayed accumulation of ADP_f _(i.e., the concentration of unbound ADP in the cytoplasm), which has been implicated in IMP and subsequently ROS formation [8,11]. This latter, in turn, may lead to oxidative stress with formation of chemical products of ROS reactions, such as oxidised glutathione and lipid hydroperoxides [12]. Despite the potential antioxidant capacity of Cr supplementation, its effects on oxidative stress and, consequently, cardiovascular parameters in experimental models of hypertension are still unknown.

This is a short-report on the effects of Cr supplementation on oxidative stress, heart structure, and arterial blood pressure in spontaneously hypertensive rats (SHR), a well-established experimental model of arterial hypertension [13].

## Material and methods

### Procedures

This study was approved by the institution's ethical committee and was conducted in accordance with the National Research Council's Guidelines for the Care and Use of Laboratory Animals. Male SHR (26 weeks old) were housed under controlled environmental conditions (22°C; 12:12-h light:dark period) with free access to commercial chow and water. Animals were randomly allocated into two groups to receive either Cr (n = 8; 5 g/kg/d) or placebo (Pl; n = 7; distillated water). The groups have similar body mass (Cr = 324.7 ± 41.9 vs. Pl = 325.2 ± 21.6; p = 0.97). Cr monohydrate was administered by gavage for nine weeks. Forty-eight hours after the intervention, arterial blood pressure and heart rate were invasively measured using a catheter inserted into the femoral artery [14]. Thereafter, animals were killed by decapitation. Plasma, heart, carotid artery, plantaris, and extensor digitorum longus (EDL) muscles were isolated, weighted and deep frozen at -80°C for further analyses. Cardiomyocyte width and cardiac collagen deposition were also assessed by histological analyses, as measures of cardiac remodeling [15]. Additionally, lipid hydroperoxidation (an important marker of oxidative stress) was determined in the plasma, heart, carotid artery, and skeletal muscles. These aforementioned methods have been described in details below.

### Hemodynamic parameters

After an intra-peritoneal anesthetic injection (80 mg/kg ketamine and 12 mg/kg xylazine, i.p.), a catheter filled with 0.06 mL of saline was inserted into the femoral artery of rats. Twenty four hours after the catheter insertion, the arterial cannula was connected to a strain-gauge transducer (Blood Pressure XDCR; Kent Scientific, Torrington, CT, USA), and arterial pressure signals were recorded over a 30 min period in conscious rats by a microcomputer equipped with an analog-to-digital converter board (WinDaq, 2 kHz, DATAQ, Springfield, OH, USA). The recorded data were analyzed on a beat-to-beat basis to quantify systolic, diastolic and mean arterial pressure, as well as heart rate.

### Histological analyses

Cardiac chambers were fixed by immersion in 4% buffered formalin and embedded in paraffin for routine histologic processing. Sections (4 μm) were stained with hematoxylin and eosin for examination by light microscopy. Only nucleated cardiac myocytes from areas of transversely cut muscle fibers were included in the analysis. Quantification of left ventricular fibrosis was achieved by Sirius red staining. Cardiac myocyte width and ventricular fibrosis were measured in the LV free wall with a computer assisted morphometric system (Leica Quantimet 500, Cambridge, UK).

### Lipid hydroperoxidation measurement

Lipid hydroperoxidation was assessed since this oxidative stress marker has been implicated in the pathogenesis of a number of cardiovascular diseases, including arterial hypertension [16,17]. Lipid hydroperoxides were evaluated by the ferrous oxidation-xylenol orange technique (FOX2) [18]. Plasma, Heart, Carotid Artery, Plantar and EDL samples were homogenized in phosphate-buffered saline (PBS; 100 mmol/L, pH 7.4) and immediately centrifuged at 12.000 g for 20 min at 4°C. The homogenate was precipitated with trichloroacetic acid (10% w/v) and centrifuged (12.000 g for 20 min at 4°C). Supernatant was mixed with FOX reagent (250 mmol/L ammonium ferrous sulfate, 100 mmol/L xylenol orange, 25 mmol/L H2SO4 and 4 mmol/L BHT in 90% methanol) and incubated at room temperature for 20 min. The absorbance of the sample was read at 560 nm in a spectrophotometer.

### Statistical analysis

Data are expressed as mean ± standard error. The dependent variables were tested by unpaired Student's *t *test. Cohen's d effect size (Cr group minus placebo group divided by the standard deviation pooled) was also calculated for dependent variables. The level of significance was previously set at p < 0.05.

## Results

As shown in Table 1, there were no significant differences in hemodynamic parameters between groups following the intervention.

**Table 1:** Hemodynamic parameters following either creatine (Cr) or placebo supplementation

Hemodynamic parameters	Placebo	Cr	Effect Size	p value
Systolic arterial blood pressure (mmHg)	203 ± 7.2	187 ± 5.8	-0.85	0.11

Diastolic arterial blood pressure (mmHg)	143 ± 5.3	130 ± 5.4	-0.82	0.12

Mean arterial blood pressure (mmHg)	172 ± 6.1	157 ± 5.8	-0.82	0.10

Heart rate (beats.min^-1^)	329 ± 14.6	323 ± 8.2	-0.18	0.73

Additionally, no significant differences between groups were shown in heart weight, cardiomyocyte width, and cardiac collagen content (Table 2). Lipid hydroperoxidation also remained unchanged in the coronary artery, heart, plasma, plantaris, and EDL (Table 3).

**Table 2:** Heart structure following either Cr or placebo supplementation

Heart structure	Placebo	Cr	Effect Size	p value
Heart weight (g)	4.0 ± 0.20	3.8 ± 0.01	0.83	0.38

Cardiomyocyte width (μm)	14.1 ± 0.4	15.1 ± 0.4	-0.86	0.13

Cardiac collagen content (%)	9.1 ± 0.6	8.5 ± 0.5	0.30	0.49

**Table 3:** Lipid hydroperoxides following either Cr or placebo supplementation

Tissue	Placebo	Cr	Effect Size	p value
Carotid artery (mmol.mg^-1 ^of total protein)	12.2 ± 1.7	12.6 ± 1.5	-0.14	0.87

Heart (mmol.mg^-1 ^of total protein)	14.6 ± 1.1	11.5 ± 1.8	0.74	0.15

Plasma (mmol.mg^-1 ^of total protein)	56.0 ± 3.2	67.7 ± 9.1	-0.76	0.19

Plantaris muscles (mmol.mg^-1 ^of total protein)	9.0 ± 0.8	10.0 ± 0.8	-0.35	0.40

EDL muscles (mmol.mg^-1 ^of total protein)	17.2 ± 1.5	14.9 ± 1.4	0.73	0.30

## Comments

Cr intake failed to attenuate oxidative stress in the cardiovascular system (i.e., heart and artery) as well in other tissues (i.e., plasma and skeletal muscle) in SHR. Furthermore, Cr did not affect either the heart structure or the hemodynamic parameters. Altogether, these data suggest that Cr supplementation does not exert therapeutically relevant effects in a model of SHR.

It has been speculated that the coupling of Cr with ATP into the mitochondria could attenuate the formation of reactive oxygen species by stimulating the respiration rate and reducing the free energy required for ATP synthesis [8]. Furthermore, Cr appears to act as a direct scavenger of radical species in face of oxidative stress [8,9]. These in vitro antioxidant proprieties confer to Cr a possible therapeutic role in diseases in which oxidative stress is exacerbated and related to pathological conditions. However, the current results were in contrast to our hypothesis. There are two potential speculations for the lack of any "positive" outcome in this study. First, the arterial blood pressure peaks at 24 weeks of age in SHR [13]. Therefore, one may assume - despite the lack of a healthy control group - that our rats displayed severe arterial hypertension. In such extreme conditions, Cr may be not capable of reverting cardiovascular dysfunction. Second, Cr metabolism is divergent among species [19], meaning that the in vitro antioxidant effects of Cr may not be extended to in vivo models. Further studies with other experimental models of hypertension as well as randomized controlled trials with humans are required to determine whether Cr supplementation can alleviate oxidative stress and cardiovascular dysfunction in arterial hypertension.

In summary, Cr supplementation did not affect oxidative stress or cardiovascular parameters in SHR model.

## Abbreviations

ATP: Adenosine triphosphate; Cr: Creatine; EDL: Extensor digitorum longus; SHR: Spontaneously hypertensive rats.

## Competing interests

The authors declare that they have no competing interests.

## Authors' contributions

CRRA was a significant writer and responsible for concept and design, experimental procedures, data analyses and interpretation. IHM, PR, HN and LRGB have participated in experimental procedures, data interpretation and manuscript preparation. AHLJ, PCB and MCI have participated in data interpretation and manuscript review. BG was a significant writer and responsible for data interpretation. All authors read and approved the final manuscript.
